# Modulation of Host miRNAs by Intracellular Bacterial Pathogens

**DOI:** 10.3389/fcimb.2016.00079

**Published:** 2016-08-03

**Authors:** Kishore Das, Omar Garnica, Subramanian Dhandayuthapani

**Affiliations:** Center of Emphasis in Infectious Diseases and Department of Biomedical Sciences, Paul L. Foster School of Medicine, Texas Tech University Health Sciences Center El PasoEl Paso, TX, USA

**Keywords:** microRNA, *Mycobacterium*, *Salmonella*, *Listeria*, *Francisella*, macrophages, host, regulation

## Abstract

MicroRNAs (miRNAs) are short non-coding RNAs that regulate the expression of protein coding genes of viruses and eukaryotes at the post-transcriptional level. The eukaryotic genes regulated by miRNAs include those whose products are critical for biological processes such as cell proliferation, metabolic pathways, immune response, and development. It is now increasingly recognized that modulation of miRNAs associated with biological processes is one of the strategies adopted by bacterial pathogens to survive inside host cells. In this review, we present an overview of the recent findings on alterations of miRNAs in the host cells by facultative intracellular bacterial pathogens. In addition, we discuss how the altered miRNAs help in the survival of these pathogens in the intracellular environment.

## Introduction

Initially discovered in the nematode *Caenorhabditis elegans* in 1993 (Lee et al., 1993), miRNAs are a non-coding class of RNAs which regulate the expression of protein coding genes post transcriptionally either by degrading mRNA or by repressing translation. They are expressed by eukaryotic and viral genomes and they range in size from 20 to 22 nucleotides in length (Bartel, 2009; Krol et al., 2010). Although their identification in different species still continues, the miRNAs already identified have shown striking sequence conservation across species and phyla, suggesting possible similarities in function among them as well. In humans, over 2500 matured miRNAs (miRBase.org) have been identified so far and it is estimated that ~60% of the human protein coding genes are regulated by miRNAs (Friedman et al., 2009). Interestingly, however, since miRNAs have the ability to interact with multiple mRNAs, the number of mRNAs regulated by a single miRNA as well as the number of miRNAs by which a single mRNA can be regulated may vary greatly (Krol et al., 2010).

A number of studies have focused on the different aspects of miRNA synthesis. miRNA is first synthesized as a primary precursor, or pri-miRNA, by RNA polymerase II (Borchert et al., 2006). Subsequently, this pri-miRNA gets processed into miRNA by two enzymes of the RNAse III family, namely Drosha and Dicer, which are located in the nucleus and in the cytoplasm, respectively. While Drosha cleaves the pri-miRNA into a 70 bp pre-miRNA hairpin structure (Lee et al., 2003), Dicer cleaves this further into a 20–22 bp miRNA duplex. This duplex consists of a guide strand and a messenger strand in which the messenger strand ultimately gets degraded. Following the degradation of the messenger strand, the guide strand becomes matured miRNA and gets loaded into the RNA induced silencing complex (RISC) which consists of Ago2 and other proteins such as Tar RNA binding protein (TRBP) and KH type splicing regulatory protein (KSRP) (Wang et al., 2009). Finally, RISC (loaded with the guide strand) binds with complementary sequences in the 3′-UTR of the target mRNA transcripts (protein coding mRNA sequences) and inhibits their translation or leads to their degradation (Bartel, 2009). The specificity of the target sequences is determined by the 6-8 nucleotide “seed region” located at the 5′-end of miRNA. Intriguingly, miRNAs are found in body fluids such as serum, plasma, saliva, milk, and urine (Weber et al., 2010). Although most of these miRNAs are bound to Ago2 and are believed to be released by dead cells (Turchinovich et al., 2011), isolation of exosome bound miRNAs suggests the possibility that they may be secreted out of the cells instead (Singh et al., 2015).

It is now well-established that miRNAs play pivotal roles in regulating cellular processes like cell proliferation, metabolic pathways, immune response, and development (Bartel, 2004; Kloosterman and Plasterk, 2006; Taganov et al., 2007; O'connell et al., 2010; Deiuliis, 2016). Thus, expression of miRNAs at normal levels is crucial for maintaining homeostasis in all eukaryotes. Most importantly, alterations in the levels of cellular miRNAs, due to either natural or unnatural causes, may have a huge impact on life. Examples of this extensive impact are genetic and epigenetic changes in the genome of humans which alter miRNAs expression. These changes not only affect cellular processes, but they also result in the manifestation of life threatening diseases (Calin and Croce, 2006; Lawrie, 2007; Saunders et al., 2007). Some serious diseases resulting from altered miRNA expression in humans are cancer, kidney failure, cardiac disease, diabetes, and liver cirrhosis (Kloosterman and Plasterk, 2006; Rome, 2013; Finch et al., 2014; Santovito et al., 2015; Trionfini et al., 2015). Notably, miRNAs released in bodily fluids serve as biomarkers for diagnosis or treatment of certain diseases (Guay and Regazzi, 2013; Mulrane et al., 2014; Pal et al., 2015; Hayes and Chayama, 2016).

Altered expression of miRNAs also occurs when pathogens infect their hosts, which includes humans, and this was first observed in viral infections (Cullen, 2011). In addition to modulating the expression of host miRNAs, several DNA viruses like Herpes simplex virus are known to use their genome encoded miRNAs to alter the expression of host mRNAs to increase their pathogenicity (Gottwein and Cullen, 2008; Umbach et al., 2008). Viruses like HIV-1 alter host miRNAs to maintain HIV-1 latency in resting CD4^+^ T cells (Swaminathan et al., 2013). With respect to bacteria, the very first evidence for miRNA alteration came from infection of a plant, *Arabidopsis thalina*, by the bacterium *Pseudomonas syringae* (Navarro et al., 2008). It was noted that *P. syringae* infection of *A. thalina* upregulated the expression of miR-393a, which in turn affected the receptors for auxin, a negative regulator of plant defense. Following this report, several groups initiated studies to assess the effects of bacterial components such as lipopolysaccharide (LPS) and lipomannan on the miRNAs of mammalian cells or hosts (Tili et al., 2007; Xiao et al., 2009; Rajaram et al., 2011). The fact that bacterial pathogens have the ability to alter the host miRNAs is now well-defined and experimentally verified by using disease causing pathogens such as *Helicobacter pylori, Listeria monocytogenes, Salmonella enterica* serovar Typhimurium, *Mycobacterium tuberculosis* and others (Eulalio et al., 2012; Harapan et al., 2013; Staedel and Darfeuille, 2013; Maudet et al., 2014a). These studies have also unraveled that host cellular miRNAs are manipulated by bacterial pathogens for their own survival. In this review, we specifically focus on the manipulation of host miRNAs by intracellular bacteria that belong to the genus *Mycobacteria, Salmonella, Listeria* and *Francisella*.

## Mycobacteria

The genus *Mycobacterium* contains several intracellular pathogens affecting humans and animals. While *M. tuberculosis* and *M. leprae* are the etiological agents for the diseases tuberculosis and leprosy in humans, *M. bovis* and *M. avium* are pathogens associated with cattle tuberculosis and respiratory illness in birds, respectively. *Bacillus Calmette-Guérin* (BCG) is an attenuated strain of *M. bovis* and is an approved vaccine against human tuberculosis (Andersen, 2001; Andersen and Doherty, 2005). All these bacteria, including the vaccine strain BCG, have the ability to survive in phagocytes. Since *M. tuberculosis* is a serious cause of morbidity and mortality, this pathogen is a relatively better studied organism than other species of *Mycobacteria*. It gains entry into macrophages or phagocytes by receptor mediated endocytosis; primarily through mannose receptors located on the surface of phagocytes (Schlesinger et al., 1994, 1996; Fenton et al., 2005). After gaining entry, the endocytosed bacteria modulate or reprogram the maturation of phagosomes using cell wall components and secreted products, a process known as “phagosome maturation arrest” (Vergne et al., 2004a,b, 2005). Essentially, the “phagosome maturation arrest” is characterized by the arrest in the recruitment of molecules required for trafficking machinery and membrane fusion which include Rab GTPases and Rab interacting factors. It was frequently observed that *M. tuberculosis* containing phagosomes acquire Rab5A GTPase but fail to acquire Rab5A effectors like EEA1 and hVPS34 (Fratti et al., 2001, 2003). As a consequence, the immature phagosomes containing *M. tuberculosis* do not fuse with lysosomes, which allow the bacteria to escape from the toxic arsenals released by the lysosomal vacuoles. *M. tuberculosis* within phagosomal compartments not only survives, but also replicates by utilizing the host's biomolecules as nutrients. In addition, *M. tuberculosis* modulates cell death pathways like apoptosis, necrosis, and autophagy to avoid the death of infected cells (Moraco and Kornfeld, 2014).

Recently, several studies have explored the role of miRNAs in mycobacterial infection (Harapan et al., 2013; Table 1). However, a connection between miRNAs and phagosomal formation/trafficking has been made in only a few of these studies. Bettencourt et al. (2013) have obtained the first evidence in this regard and reported that *M. tuberculosis* infection induces the expression of miR-142-3p in murine J774A.1 cells and in primary human macrophages leading to the down regulation of N-Wasp, an actin binding protein. The down regulation of N-Wasp limits the amount of actin available for the formation of actin filament in the early phagosome, thereby reducing the uptake of *M. tuberculosis* pathogen. This was experimentally confirmed by siRNA mediated knock down of N-Wasp in human macrophages, indicating that miR-142-3p is one of the key miRNAs modulated by mycobacteria to gain entry into macrophages. Additionally, this observation gains support from previous studies which report that virulent *M. tuberculosis* blocks actin filament assembly to facilitate its survival inside the host cell (Anes et al., 2003).

**Table 1:** MicroRNAs altered by mycobacterial species or their proteins^*^.

**Function**	**miRNA**	**Target**	**Molecule altered**	**Cell /Tissue examined**	**Bacterial Species/proteins**	**References**
Phagocytosis	miR-142-3p	N-Wasp	Actin	J774A.1 and primary human macrophages	*M. tuberculosis*	Bettencourt et al., 2013
Phagosomal maturation	miR-15a miR-21-3p miR-22-3P miR-23a miR-30b-5p miR-142-5p	Rab family of proteins	Rab family of proteins	Bovine alveolar macrophages	*M. bovis*	Vegh et al., 2015
NO Suppression	miR-146a	NF-κB, MAPK	TRAF6	RAW264.7 macrophages and BMDM	*BCG*	Li et al., 2016
	miR-155	C/EBPβ	NO synthesis	RAW264.7 macrophages	*M. marinum*	Qin et al., 2016
Apoptosis	let-7e miR-29a	Caspase 3 Caspase 7	Caspase 3 Caspase 7	Human monocyte derived macrophages	*M. avium*	Sharbati et al., 2011
	miR-21	NF-κB	Bcl-2	RAW264.7 macrophages	*M. tuberculosis* MPT64	Wang Q. et al., 2014
	miR-155	FOXO3		Human monocytes, THP-1 cells	*M. tuberculosis*	Huang et al., 2015
	miR-582-5p	FOXO1		Human monocytes	*M. tuberculosis*	Liu et al., 2013
Autophagy	miR-17-5p	ULK-1	ULK-1	RAW264.7 macrophages	*BCG*	Duan et al., 2015
	miR-17-5p	Mcl-1/STAT3	Beclin-1	RAW264.7 macrophages and BMDM	*M. tuberculosis*	Kumar et al., 2016
	miR-30a		Beclin-1	Human monocytes	*M. tuberculosis*	Chen et al., 2015
	miR-33 miR-33^*^	ATG5, LAMP1	ATG5, ATG12, LAMP1, LC3B, AMPK, FOXO3 and TEEB	Mouse peritoneal and BMDM	*M. tuberculosis*	Ouimet et al., 2016
	miR-125a-3p	UVRAG		RAW264.7 macrophages and BMDM	*M. tuberculosis*	Kim et al., 2015
	miR-3619-5p	Cathepsin S	Cathepsin S	THP-1 macrophages	*BCG*	Pawar et al., 2016
Signaling	Let-7f	A20	NF-κB	RAW264.7 macrophages and BMDM	*M. tuberculosis* ESAT-6	Kumar et al., 2015
	miR-26a, miR-132	P300	IFN-γ	Human primary macrophages	*M. tuberculosis*	Ni et al., 2014
	miR-124	TLR-6, MyD88, TNFR6, TNF-α	TLR-6, MyD88, TNFR-AF6, TNF-α	Human peripheral leukocytes	*M. tuberculosis*	Ma et al., 2014a
				Murine alveolar macrophages, RAW264.7 macrophages, A549 epithelial cells	*BCG*	Ma et al., 2014a,b
	miR-146a	IRAK-1,TRAF-6	TNF-α, IL-1β, IL-6 and MCP-1	RAW264.7 macrophages	*BCG*	Li et al., 2013
	miR-149	MYD-88	Inflammatory mediators	RAW264.7 macrophages	*BCG*	Xu et al., 2014
	miR-155	SHIP1, SOCS1, FADD, C/EBP- β, NF-κB	Inflammatory mediators, cytokines	Macrophages	*M. tuberculosis*	Rajaram et al., 2011
				RAW264.7 and mouse BMDM	*M. tuberculosis* ESAT-6	Kumar et al., 2012
				Mouse peritoneal macrophages	*BCG*	Ghorpade et al., 2012
				RAW264.7 macrophages	*BCG*	Wang J. et al., 2014
	miR-223	IKKα subunit of NF-κB	CCL3, CXCL2 and IL-6	Human Blood and Lung specimen	*M. tuberculosis*	Dorhoi et al., 2013
Cytokines	miR-26a	IFN-γ	IFN-γ	Human macrophages	*M. tuberculosis*	Ni et al., 2014
	miR-29	IFN-γ	IFN-γ	Mouse CD4^+^ and CD8^+^ T cells	*BCG*	Ma et al., 2011
	miR-99b	Genes for TNF-α and TNFRSF-4 receptors	TNF-α and TNFRSF-4	Dendritic cells and macrophages	*M. tuberculosis*	Singh et al., 2013
	miR-125b	TNF-α	TNF-α	Macrophages	*M. tuberculosis*	Rajaram et al., 2011
	miR-132	P300	IFN-γ	Human macrophages	*M. tuberculosis*	Ni et al., 2014
	miR-144^*^		IFN-γ, TNF-α	Human blood	*M. tuberculosis*	Liu et al., 2011
	miR-206	TIMP3	Cytokines, MMP	THP-1 macrophage	*M. tuberculosis*	Fu et al., 2016
Antimicrobial peptides	miR-21			Human monocytes Biopsy from Leprosy patients	*M. leprae*	Liu et al., 2012
Antigen presentation	miR-381-3p	CD1c	T cell responses	Human dendritic cells	*BCG*	Wen et al., 2016

* Indicates that only miRNAs with known functions are listed.

In addition to the uptake process, miRNAs involvement in endo-lysosomal pathways of mycobacteria has also surfaced. This includes miRNAs miR-15a, miR-21-3p, miR-22-3P, miR-23a, miR-30b-5p, and miR142-5p which get upregulated in bovine alveolar macrophages in response to *M. bovis* infection (Vegh et al., 2015). It was predicted that these miRNAs target genes of the Rab family of membrane trafficking proteins like Rab4a, Rab5b, Rab5c, and Rab7a, Rab11, and Rab22a and modulate them to prevent phagosome maturation, thus enabling mycobacteria to survive within phagosomes. As noted, a key feature of mycobacterial phagosomes is the acquisition of the early endosomal marker Rab5 and lack of acquisition of late endosomal marker Rab7 (Via et al., 1997). An additional feature of mycobacterial phagosomes is the recruitment of copious amounts of Rab22a as compared to other phagosomes (Roberts et al., 2006). As the miRNAs reported by Vegh et al. target all of the important Rab proteins associated with mycobacterial phagosomes, future studies assessing the relationship between the above miRNAs and Rab genes may provide valuable insights into the role of miRNAs in the prevention of mycobacterial phagosome maturation.

Furthermore, evidence that mycobacteria modulate miRNAs to prevent apoptosis is also presented. To inhibit apoptosis, *M. tuberculosis* upregulates the expression of miR-582-5p and miR-155 and both of these miRNAs showed elevated expression in the monocytes of tuberculosis patients in comparison to healthy controls. *In vitro* reporter assays confirmed that miR-582-5p and miR-155 down regulate the transcription factors FOXO1 and FOXO3, respectively, to inhibit apoptosis in these cells (Liu et al., 2013; Huang et al., 2015). An *M. tuberculosis* secretory protein MPT64 has also been implicated in the prevention of apoptosis by acting upon Bcl2 through miR-21(Wang Q. et al., 2014). *M. avium*, on the other hand, upregulates miRNAs let-7e and miR-29a in human monocyte derived macrophages with concomitant decrease in the expression of apoptosis associated proteins caspase 3 and caspase 7 (Sharbati et al., 2011). Reporter assays again revealed that let-7e and miR-29a target mRNA of caspase 3 and caspase 7, respectively, to down regulate their expression (Sharbati et al., 2011).

Although autophagy is primarily a cellular recycling process, emerging evidences suggest that it is also a cellular defense mechanism (Jo et al., 2013). *M. tuberculosis* partly evades autophagy using the secretory proteins ESAT-6 and CFP-10 encoded by the ESX1 locus (Zhang et al., 2012) and the cell wall lipid lipoarbinomannan (LAM) (Shui et al., 2011). Recent studies show that *M. tuberculosis* modulates multiple miRNAs to evade autophagy of infected macrophages. Duan et al. (2015) have reported that BCG prevents the formation of autophagosomes by altering the expression of miR-17-5p. RAW264.7 macrophages infected with BCG upregulates the expression of miR-17-5p which in turn leads to the down regulation of its target, ULK, a protein which regulates autophagosome formation. In contrast, Kumar et al. (2016) have reported that *M. tuberculosis* downregulates the expression of miR-17-5p in infected macrophages and this is accompanied by upregulation of its target proteins Mcl-1 and STAT3, a transcriptional regulator of Mcl-1. Mcl-1 in turn interacts with beclin-1 to inhibit autophagy and evidence in this regard has been presented (Kumar et al., 2016). It is not known, however, why BCG upregulates and *M. tuberculosis* downregulates miR-17-5p in macrophages to prevent autophagy. On the other hand, Kim et al. (2015) have noticed upregulation of miRNA-125a-3p in *M. tuberculosis* infected macrophages. MiRNA-125a-3p targets UV radiation resistance-associated gene (UVRAG) in order to prevent autophagy and this was experimentally verified by overexpressing miR-125a-3p or UVRAG protein in macrophages and using inhibitors against miR125a-3p. A third miRNA implicated in the inhibition of autophagy by *M. tuberculosis* is miR-30a and this was predicted to act upon beclin-1 since monocytes isolated from tuberculosis patients exhibited a negative correlation between the concentrations of miR-30a and beclin-1 (Chen et al., 2015). Incidentally, we have shown that miRNA-30a is differentially expressed in THP-1 cells infected with *M. tuberculosis* (Das et al., 2013). Further, a recent study has noticed that *M. tuberculosis* upregulates miR-33 and its precursor miR-33^*^ in macrophages to inhibit autophagy (Ouimet et al., 2016). Upregulation of miR-33 and miR-33^*^ leads to the repression of several key autophagy effector molecules such as ATG5, ATG12, LAMP1, LC3B, AMPK, and FOXO3 and this was verified by silencing of miR-33 and miR-33^*^ by genetic and pharmacological means; thus suggesting that miR-33 inhibition is an important pathway to prevent autophagy by *M. tuberculosis*. Another study has reported (Pawar et al., 2016) that BCG downregulates the expression of miR-3619-5p leading to the upregulation of its target protein cathepsin S (CTSS), which is a lysosmal cysteine protease. It has been observed that inhibition of CTSS expression enhances autophagy in different cells (Zhang L. et al., 2014) and it is likely, therefore, that upregulation of CTSS by BCG through miR-3619-5p could prevent autophagy.

*Mycobacteria* also modulate miRNAs associated with signaling pathways which enhance their survival inside hosts. MiR-155 targets multiple proteins such as SHIP1, SOCS1, FADD, and C/EBP-β in the innate immune signaling pathways and alters the expression of inflammatory mediators. Its expression is upregulated in macrophages upon infection by both *M. tuberculosis* and BCG (Rajaram et al., 2011; Sharbati et al., 2011; Ghorpade et al., 2012; Kumar et al., 2012; Wang et al., 2013; Wang J. et al., 2014). Although this upregulation occurs primarily due to the sensing of the pathogens by TLRs of macrophages, the secreted ESAT-6 protein of *M. tuberculosis* also seems to contribute to the induction of miR-155 as *M. tuberculosis* mutant strain lacking in ESAT-6 has shown relatively less induction of miR-155 in macrophages than the wild type strain (Kumar et al., 2012). According to Kumar et al. the inhibition of miR-155 in RAW264.7 and murine bone marrow derived macrophages (BMDM) affects the survival of *M. tuberculosis* in these cells. In contrast, Ghorpade et al. (2012) have noted that BCG mediated upregulation of miR-155 leads to apoptosis of the infected cells through NF-κB signaling. Upregulated miR-155 has also been noticed to be detrimental for mycobacteria as it induces autophagy by repressing the Rheb and mTOR signaling pathways (Wang et al., 2013). Furthermore, miR-155 upregulation has been shown to increase the synthesis of TNF-α through the SHIP1 pathway (Rajaram et al., 2011). Considering all these negative effects, it remains highly unexplainable how miR155's function benefits the survival of mycobacteria inside the host. Nevertheless, it appears that mycobacteria do have mechanisms to counter the negative effects of miR-155 and make it beneficial for their survival. For instance, lipomannan of the cell wall of *M. tuberculosis* can inhibit TNF-α synthesis and counteract the effect of miR-155 upregulation (Rajaram et al., 2011). Additionally, *M. tuberculosis* has been shown to induce miR-125b which directly targets the mRNA of TNF-α (Rajaram et al., 2011). This can reduce TNF-α synthesis and balance the effect of miR-155 upregulation.

Recently, Kumar et al. (2015) have found that *M. tuberculosis* down regulates the expression of miRNA let-7f, which targets mRNA of A20, an inhibitor of NF-κB. Significantly, the down regulation of let-7f is accompanied by concomitant upregulation of A20 in mice infected with *M. tuberculosis* (Kumar et al., 2015). Additionally, *M. tuberculosis* fails to survive in macrophages deficient in A20. These observations provide strong evidence that *M. tuberculosis* uses let-7f to increase its survival inside the host by inhibiting NF-κB through the expression of A20. This is highlighted by the additional observation that down regulation of let-7f is dependent upon ESAT-6, which is a major virulence factor of *M. tuberculosis*. In addition, mycobacterial infections of macrophages were also found to induce significant levels of miR-146a expression in a time and dose-dependent manner (Li et al., 2013). This miRNA targets two key molecules involved in the TLR/NF-κB signaling pathway cascades: interleukin-1 receptor-associated kinase-1 (IRAK-1) and TNF receptor-associated factor-6 (TRAF-6). It is likely that the increased expression of miR-146a during *M. tuberculosis* infection will affect the TLR/NF-κB and IRAK-1 pathways and as a consequence the induction of proinflammatory cytokines TNF-α, IL-1β, IL-6, and chemokine MCP-1 will be reduced. Another miRNA that behaves in a similar fashion as miR-146a is miR-223 which modulates the IKKα subunit of NF-κB and regulates the inflammatory responses in mononuclear phagocytes. MiR-223 is significantly upregulated in the blood and lungs of tuberculosis patients and in the blood of mice infected with *M. tuberculosis* (Dorhoi et al., 2013). Also, it appears that *M. tuberculosis* suppresses CCL3, CXCL2, and IL-6 by upregulating the levels of miR-223 as these are its direct targets. Lastly, it was also noticed that a knockout mouse lacking in miR-223 (*miR-223* −*/*−) was found to be susceptible to *M. tuberculosis* infection (Dorhoi et al., 2013).

It is well-known that IFN-γ plays a critical role in resisting intracellular infection. BCG regulates IFN-γ in mouse CD4^+^ and CD8^+^ T cells by downregulating the expression of miR-29 (Ma et al., 2011) and *M. tuberculosis* regulates IFN-γ in human macrophages by upregulating the expression of miR-26a and miR-132 (Ni et al., 2014). Although, miR-29 targets IFN-γ directly, miR-26a and miR-132 target IFN-γ via P300, a transcription mediator. However, there is a significant difference between the regulation of IFN-γ by miR-29 and miR26a/132. Whereas down regulation of miR-29 leads to increased expression of IFN-γ levels in CD4^+^ and CD8^+^ T cells, upregulation of miR-26a/132 leads to the repression of IFN-γ levels. The repression of IFN-γ in CD4^+^ and CD8^+^ T cells by virulent *M. tuberculosis* may be a strategy for its survival inside the host. Further, the differential regulation of IFN-γ by *M. tuberculosis* and BCG, through different miRNAs, may reflect the differences in their genes/proteins contents. On the other hand, *M. tuberculosis* controls the levels of TNF-α in dendritic cells and macrophages through miR-99b. Both dendritic cells and macrophages infected with *M. tuberculosis* show upregulation of miR-99b and experimental down regulation of this miRNA affects the growth of *M. tuberculosis* in these cells (Singh et al., 2013). Further, inhibition of miR-99b augment the production of TNF-α and TNFRSF-4 in these cells, suggesting that miR-99b targets TNF-α and TNFRSF-4 receptor genes to control the production of TNF-α and TNFRSF-4. Overall, this emphasizes that upregulation of miR-99b by *M. tuberculosis* is critical for its survival inside host cells. Additionally, *M. tuberculosis* regulates IFN-γ and TNF-α in human T cells through miR-144^*^ and it is upregulated in the blood samples of TB patients. *In vitro* transfection studies of T-cells with miR-144^*^ indicated that this miRNA could alter the levels of IFN-γ and TNF-α production (Liu et al., 2011), suggesting that miR-144^*^ is associated with anti-TB immunity. Furthermore, it was also noticed that the expression of miR-206 is markedly upregulated in *M. tuberculosis* infected THP-1 cells and that upregulated miR-206 positively regulates the inflammatory cytokines IL-1β, IL-6, IFN-γ, TNF-α, and MMP9 (Fu et al., 2016). This may indicate that miR-206 is the key regulator of inflammation during *M. tuberculosis* infection and may be a potential therapeutic target.

*Mycobacteria* can also modulate toll-like receptors (TLRs) through miRNAs to reduce proinflammatory responses. MiR-124, which directly controls TLR6, myeloid differentiation factor 88 (MyD88), TNFR-associated factor 6 and TNF-α, is upregulated in patients with pulmonary tuberculosis (Ma et al., 2014a). Its expression is also elevated in BCG infected murine alveolar macrophages, RAW264.7 macrophage cell line (Ma et al., 2014a), and alveolar epithelial cell line A549 (Ma et al., 2014b). Therefore, the upregulation of miR-124 in tuberculosis patients and in the cells may be associated with regulation of proinflammatory responses. However, BCG dynamically reduces the expression of miR-149 in RAW264.7 cells with simultaneous increase in the expression of MyD88 (Xu et al., 2014). Using a luciferase reporter assay and immunoblotting against MyD88, this study identified that miR-149 directly targets the 3′-UTR of MyD88 mRNA and BCG mediated increase in MyD88 expression is linked to the increased production of inflammatory mediators NF-κB1, TNF-α, and IL-6. Lastly, a recent study has noted that BCG downregulates TLR-2 activated signaling events by upregulating the expression of Sonic Hedgehog (SHH) receptors (Ghorpade et al., 2013). This study speculates that this effect could be due to miR-31 and miR-150 which target MyD88, an adaptor protein of TLR2 signaling.

In order to kill invading pathogens, macrophages and other phagocytes generate superoxide (O_2_^−^) and nitric oxide (NO) through membrane bound NADPH oxidase (NOX2) and inducible nitric acid synthase (iNOS2), respectively (Ehrt and Schnappinger, 2009). *Mycobacteria* avoid killing by these reactive species by internalizing through specific receptors (Schlesinger et al., 2008). In particular, *M. tuberculosis* seems to use mannose receptors to escape from the bactericidal effects of superoxide (Astarie-Dequeker et al., 1999). Although no mycobacteria altered miRNA is implicated in the modulation of O_2_^−^ generation in macrophages, a recent study has reported that BCG can alter NO synthesis in macrophages through miR-146a (Li et al., 2016). It was observed that miR-146a expression in RAW264.7 and mouse BMDM is induced following infection with BCG in a dose dependent manner, which in turn suppresses the expression of iNOS indirectly by silencing TRAF-6 mRNA. Further, inhibition of endogenous miR-146a was noticed to enhance the NO production and consequently BCG clearance, thus providing a direct a relationship between miR-146a induction and BCG survival in macrophages. It is worth noting here that this is the first report to link the modulation of oxidative stress in host cells by a bacterium through miRNA. Additionally, it has been reported that *M. marinum* survival in macrophages is increased by miR-155 mediated suppression of NO (Qin et al., 2016).

CD1c is an important lipid antigen presenting glycoprotein in dendritic cells (DCs). Studies have already reported that CD1c levels in *M. tuberculosis* infected DCs cells are greatly reduced and this prevents the presentation of *Mtb* antigens to T cells (Stenger et al., 1998; Gagliardi et al., 2007). A recent study has unveiled that *M. tuberculosis* causes this by upregulating the expression of miR-381-3p (Wen et al., 2016) and this study has noted that DCs of TB patients had elevated expression of miR-381-3p and this had an inverse relationship with CD1c. This relationship was further explored *in vitro* by infecting DCs with BCG which not only complemented the findings in TB patients, but also revealed that suppression of antigen presentation in BCG infected DCs could be reversed by inhibiting miR-381-3p. These results may imply that miRNAs can be exploited in designing future vaccines against TB.

*M. leprae* is a difficult organism to study because of its non-cultivability under laboratory conditions. Nevertheless, a study has reported differentially expressed miRNAs in the skin lesions from tuberculoid leprosy and lepromatous leprosy (Liu et al., 2012). This study has noticed that miR-21 was upregulated in the skin lesions of lepromatous leprosy and in monocytes infected with *M. leprae*. MiR-21 suppresses the expression of vitamin D-dependent antimicrobial peptides CAMP and DEFB4 and upregulation of miR-21 was thought to help *M. leprae* evade antimicrobial response. Overall, *Mycobacteria* alter multiple miRNAs to modulate the host response to infections.

## Salmonella

*S. enterica* serovar Typhimurium is a food-borne pathogen of both animals and humans (Cossart and Sansonetti, 2004) that causes the disease gastroenteritis. *Salmonella* invades gastrointestinal epithelial cells, reaches the submucosa through transcytosis, and gets phagocytosed by phagocytes of the submucosal region. In intestinal epithelial cells and in phagocytes, *Salmonella* survives intracellularly in the so-called *Salmonella* containing vacuoles (SCVs) (Francis et al., 1993; Garcia-Del Portillo and Finlay, 1994). In general, *Salmonella* pathogenicity islands (SPIs) play critical roles in invasion, internalization, and survival of *Salmonella* inside host cells. Although *Salmonella* possesses five (SPI-1 to SPI-5) SPIs, SPI-1 contains the genes needed to encode the effector proteins required for the invasion of *Salmonella* to epithelial cells (Fabrega and Vila, 2013). SPI-1 also has genes to code for proteins associated with a type III secretion system (T3SS) which actually translocate the effector proteins into the host cells. Proteins encoded by SPI-2 and the virulence plasmid pSLT are essential for the survival of *Salmonella* within SCVs. Similar to that of *M. tuberculosis* containing phagosomes, the maturation and trafficking of SCVs are arrested by the effector molecules released by the *Salmonella* within SCVs. Two effector proteins SigD and SsaB seem to play pivotal roles in this process. Whereas SigD modulates phosphoinositides of the membrane to arrest vesicular trafficking (Hernandez et al., 2004), SsaB inactivates the Hook3 component of the endocytic vesicle which eventually disrupts the fusion of SCVs with lysosomes (Uchiya et al., 1999).

*In vitro* studies indicate that *Salmonella* modulates miRNAs in both epithelial cells and macrophages (Schulte et al.; Table 2). It was noticed that let-7a was downregulated in *Salmonella* infected RAW264.7 and HeLa cells and miR-21, miR-146, and miR-155 were upregulated in RAW264.7 cells. Intriguingly, none of these miRNAs showed any association with *Salmonella*'s invasion, intracellular replication, or both, as mutant *Salmonella* strains for these phenotypes had no effect on these miRNAs. It is not clear that miRNAs specific for invasion and intracellular survival of *Salmonella* do not exist or remain to be identified. The repression of let-7a was found to be triggered by lipopolysaccharide (LPS), through TLR4 sensing, to relieve its targets IL-6 and IL-10 from negative posttranscriptional control. Both IL-6 and IL-10 are associated with pro- and anti-inflammatory responses, respectively, (Klimpel et al., 1995) and they more than likely play a role in balancing the inflammatory response.

**Table 2:** MicroRNAs altered by Salmonella species^*^.

**Function**	**miRNA**	**Target**	**Molecule altered**	**Cell /Tissue examined**	**Bacterial Species**	**References**
Signaling	miR-155	SHIP1, SOCS1, FADD, C/EBP- β, NF-κB	Inflammatory mediators, cytokines	RAW264.7 macrophages, HeLa epithelial cells	*S. typhimurium*	Schulte et al., 2011
Cytokines	Let-7a	IL-6 and IL-10	IL-6 and IL-10	RAW264.7 macrophages, HeLa epithelial cells	*S. typhimurium*	Schulte et al., 2011
Cell cycle	miR-15 family	Cyclin	Cyclin	HeLa cells	*S. typhimurium*	Maudet et al., 2014b
Posttranslational Modification	miR-30c and miR-30e	Ubc-9	Ubc-9	J774A.1 macrophages	*S. typhimurium*	Verma et al., 2015
Cytoskeletal structures	miR-29A	Caveolin-2	Rho GTPase CDC42	Piglet ileal samples	*S. typhimurium*	Hoeke et al., 2013
	miR-128		M-CSF	Mouse intestinal tissue	*S. typhimurium*	Zhang T. et al., 2014
Iron homeostasis	miR-214	SLC11A1, PIGE-108A11.3	SLC11A1, PIGE-108A11.3	Whole blood of piglets	*S. typhimurium*	Bao et al., 2015
Rho GTPase activation	miR-231-3p	VAV2	VAV2	Whole blood of piglets	*S. typhimurium*	Bao et al., 2015

* Indicates that only miRNAs with known functions are listed.

Recently, Maudet et al. (2014b), using a library of miRNA mimics, has noticed that *Salmonella* downregulates the expression of miR-15 family in HeLa cells. These miRNAs target Cyclin D1 protein associated with cell cycle and down regulation of miR-15 family derepresses the expression of Cyclin-1. This renders the cells to remain in G1/S phase, a growth phase that favors intracellular replication of *Salmonella*. In addition to miR-15, miR-30c, and miR-30e have also been implicated in the intracellular survival of *Salmonella* (Verma et al., 2015). Both of these miRNAs target Ubc-9 protein associated with SUMOylation, a posttranslational modification required for fundamental cellular processes.

Using the piglet model, Hoeke et al. (2013) have demonstrated that miR-29a was upregulated in ileal samples. This upregulation led to the down regulation of its target, caveolin-2, an inhibitor of Rho GTPase CDC42 that is associated with cytoskeletal structures. Experimental down regulation of caveolin-2 increased the uptake of *Salmonella* inside the cells, suggesting that modulation of miR-29a is critical for *Salmonella* infection. On the other hand, whole blood of piglets infected with *Salmonella* has shown up- and down regulation of several miRNAs of which two miRNAs have been characterized (Bao et al., 2015). These two miRNAs, miR-124 and miR-331-3p, target genes *SLC11A1* and *PIGE-108A11.3* and *VAV2*, respectively, and products of these genes are associated with the regulation of immune responses. Moreover, *Salmonella* infection upregulates the expression of miR-128 in mouse intestinal tissue. It targets macrophage colony stimulating factor (M-CSF) to downregulate its expression, which in turn leads to decreased recruitment of macrophages to clear *Salmonella* infection (Zhang T. et al., 2014). A few other studies have reported altered miRNAs expression following *Salmonella* infection (Sharbati et al., 2012; Yao et al., 2015; Uribe et al., 2016) but their roles in immune response and other functions is unclear. Interestingly, there are no reports on the subversion of miRNAs associated with the prevention of the apoptosis pathway, although *Salmonella* does have effector proteins that prevent apoptosis of infected epithelial cells at an early stage (Fabrega and Vila, 2013).

## Listeria

*L. monocytogenes* is another food-borne intracellular pathogen that causes listeriosis in humans, a disease characterized by severe septicemia. Infection of *Listeria* in pregnant women results in abortion or meningoencephalitis in the new born (Allerberger and Wagner, 2010). *Listeria* has the ability, like *Salmonella*, to survive and replicate in both phagocytic and non-phagocytic cells (Pizarro-Cerdá et al., 2012). It uses scavenger receptors for gaining entry into macrophages and receptors such as E cadherin, Met, and C1qR for internalizing into the epithelial cells. The surface proteins internalin A (InlA) and internalin B (InlB) serve as ligands to host cell receptors and the ligand-receptor interaction mediates the uptake of the bacteria. In addition, *Listeria* secretes a cholesterol dependent cytolysin called listeriolysin O (LLO) which has a dual function of inhibiting the phagosome maturation and making pores in the phagosomal membrane (Henry et al., 2006; Shaughnessy et al., 2006). Using LLO, along with two lipases PI-PLC and PC-PLC, *Listeria* breaks the phagosomal membrane and escapes into the cytosol (Smith et al., 1995). It replicates in the cytosol and becomes motile by utilizing host actin cytoskeletal machinery. A bacterial surface protein, ActA, induces the assembly of actin to give it a tail shape, called actin comet tail, and the actin based motility enables the bacterium to spread to neighboring cells (Lambrechts et al., 2008).

Schnitger et al. (2011) infected macrophages with wild type and LLO deficient *L. monoctytogenes* in parallel and analyzed the expression of miRNAs in the infected cells to identify the host miRNAs subverted by *Listeria* (Table 3). Although the infection of macrophages by *Listeria* upregulated the expression of five miRNAs, which include miR-155, miR-146a, miR-125a-3p/5p, and miR-149, surprisingly the upregulated miRNAs remained similar with both wild type and LLO deficient *L. monocytogenes*, suggesting that miRNAs only play a limited regulatory role in the inhibition of maturation of listeria containing phagosomes or in the actin tail assembly in the infected cells. Infection of intestinal epithelial Caco-2 cells with these strains also exhibited alteration in the expression of miRNAs by both strains (Izar et al., 2012). In this case, miR-146b, miR-16, and miR-155 were upregulated and let-7a1 and miR-145 were down regulated. Interestingly, some of these miRNAs were also found to be upregulated by treating the cells with purified LLO alone. However, the miR-155 was down regulated in epithelial cells infected with *L. monocytogenes* deficient in internalin A and B as compared to wild type infected cells, possibly suggesting that this strain could induce less inflammatory response in the host.

**Table 3:** MicroRNAs altered by Listeria^*^.

**Putative Function**	**miRNA**	**Target**	**Molecule altered**	**Cell /Tissue examined**	**Bacterial Species**	**Reference**
Signaling	miR-146a	IRAK-1, IRAK-2 TRAF-6	TNF-α, IL-1β, IL-6 and MCP-1	Macrophages	*L. monocytogenes*	Schnitger et al., 2011
	miR-155	SHIP1, SOCS1, FADD, C/EBP- β, NF-κB	Inflammatory mediators, cytokines	Macrophages	*L. monocytogenes*	Schnitger et al., 2011
Cytokines	miR-223	IKKα subunit of NF-κB	CCL3, CXCL2 and IL-6	Macrophages	*L. monocytogenes*	Schnitger et al., 2011
	miR-29	IFN-γ	IFN-γ	Macrophages	*L. monocytogenes*	Ma et al., 2011

* indicates that only miRNAs with known functions are listed.

Oral infection of mice with *L. monocytogenes* also showed modulation of miRNAs. One study has observed that three miRNAs (miR-192, miR-200b, and miR-215) were repressed in the intestinal tissues after orally infecting humanized mice with *L. monocytogenes* (Archambaud et al., 2012). But in conventional mice, *Listeria* infection decreased the expression of 6 miRNAs in which five (miR-143, miR-148a, miR-200b, miR-200c, and miR-378) showed variations in expression in response to the presence or absence of microbiota in the gut (Archambaud et al., 2013). In addition, it was noticed that systemic infection of *L. monocytogenes* down regulated the expression of miR-29 in natural killer cells (NK cells) (Ma et al., 2011). MiR-29 represses INF-γ expression and its down regulation is expected to increase INF-γ levels in the host leading to resistance to infection. It is not clear, however, how *L. monocytogenes* circumvents the effect of INF-γ and survives inside the host.

## Francisella

*Francisella* are gram negative cocci and facultative intracellular pathogens. *Francisella tularensis* is the most virulent species and the causative agent of “tularensis” in humans. Other species in this genus like *F. novicida* and *F. philomiragia* are non-pathogenic species and infect only immunocompromised individuals. *Francisella* uses multiple receptors to gain entry into macrophages (Celli and Zahrt, 2013). Following uptake, the phagosomes containing *Francisella* acquires maturation markers such as CD63, LAMP-1 and Rab7 but yet fail to fuse with lysosomes (Clemens et al., 2004; Santic et al., 2005a,b; Chong et al., 2008; Wehrly et al., 2009). The bacteria later escape from the phagosomal vacuole to the cytoplasm via unknown mechanisms, possibly by disrupting the membrane of the phagosomes. It is believed that the phagosomal escape is necessary for multiplication of the bacterium in the cytosol. The intracellular events of *Francisella* are controlled by effectors released by type VI secretion system located in the *Francisella* pathogenicity island (FPI) (Santic et al., 2005b; Barker et al., 2009; Wehrly et al., 2009).

Since *F. novicida* has the ability to down regulate the expression of SHIP, the known target of miR-155, in monocytes and macrophages, Cremer et al. speculated that there might be a relationship between miR-155 and SHIP during *Francisella* infection (Cremer et al., 2009). Using a luciferase reporter assay, they were in fact able to demonstrate the upregulation of miR-155 and down regulation of SHIP in macrophages following *F. novicida* infection. It was also observed that virulent *F. tularensis* (A SCHU S4 strain) infected cells had relatively lower upregulation of miR-155 than the non-virulent *F. novicida* infected cells. This difference was attributed to the virulent strain's ability to suppress proinflammatory responses. Nevertheless, the upregulation of miR-155 was later demonstrated to be due to soluble factors released from *Francisella* (Cremer et al., 2012). A recent study using *F. tularensis* live vaccine strain (LVS) has reported that nine miRNAs were upregulated and one was down regulated in macrophages (Bandyopadhyay et al., 2014). MiR-155 is one of the upregulated miRNAs in this study for which the function is known. The roles of other upregulated miRNAs in the pathogenesis of *Francisella* remain to be investigated.

## Conclusion

**Figure 1:**
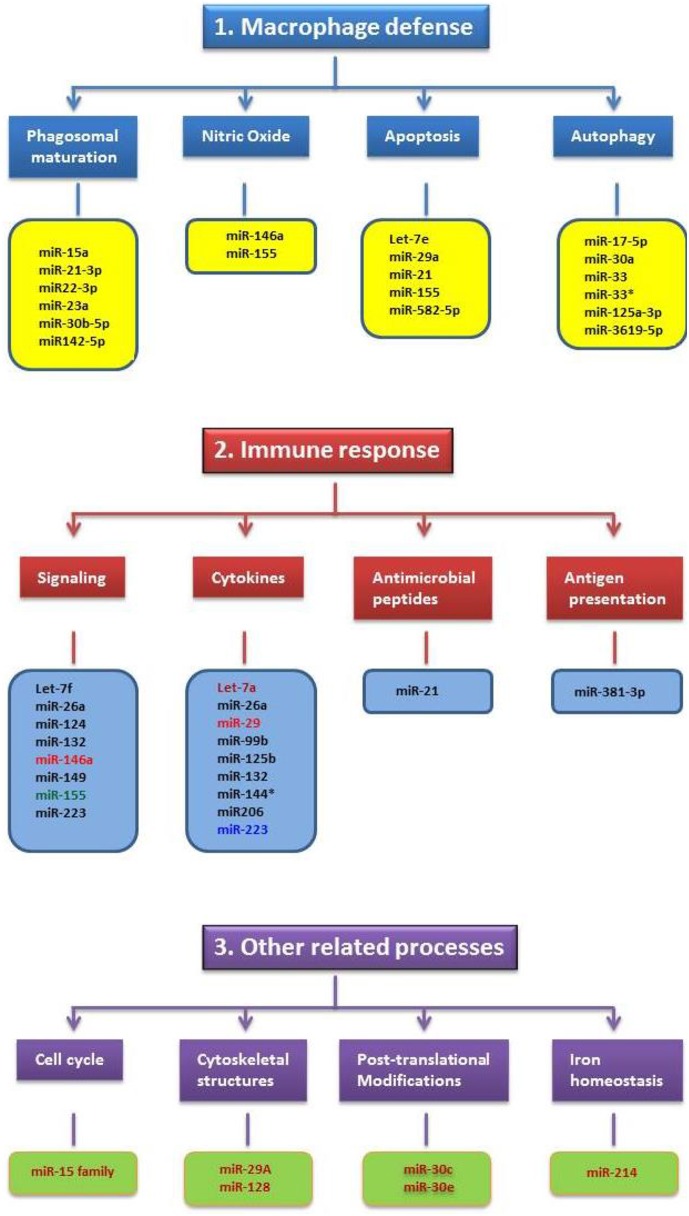
**Schematic illustrating the miRNAs altered by *Mycobacteria, Salmonella, Listeria* and *Francisella* in relation to their cellular functions**. The functions are grouped under three major categories such as macrophage defense, immune response and other related processes. Only very few miRNAs altered by these pathogens have shown common cellular functions and this includes miR-155. MiRNAs altered by specific pathogen(s) and their color(s) are as follows: Black-*Mycobacteria*; Blue-Listeria; Light Red- *Mycobacteria* and *Listeria*; Deep Red-*Salmonella*; Green-*Mycobacteria, Salmonella, Listeria and Francisella*.

Intracellular bacteria that can survive and replicate in phagocytic cells are generally considered more sophisticated than other pathogens since they have the ability to modulate the defense cells of the immune system and make them their home for survival and multiplication. Therefore, we speculated that these pathogens could alter a large number of miRNAs in infected cells as compared to non-intracellular pathogenic bacteria. Unfortunately, all four intracellular bacteria species focused here have shown only a limited number of altered miRNAs in the infected cells or in the hosts (Figure 1). The number of altered miRNAs by these bacteria in macrophages remain more or less similar to the number of miRNAs in non-phagocytic cells following *H. pylori* infection (Matsushima et al., 2011). Moreover, the miRNAs altered by intracellular pathogens show little relationship toward phagosomal maturation and trafficking. In particular, none of the miRNAs up- or down regulated with *Salmonella* or *Listeria* infections of macrophages are related to phagosome maturation (Figure 1). This is very surprising because it does not correlate with the multiple effectors employed by these pathogens to alter phagosomal maturation and trafficking. At present it is difficult to provide a meaningful explanation as to why these pathogens and their products do not alter the miRNAs associated with the phagosome maturation process or pathways associated with other bactericidal mechanisms of phagocytes. One explanation is that the effectors of these pathogens do not need regulation by miRNAs as they already have the capacity to directly hijack host molecules. The other explanation may be that the altered miRNAs may have the capacity; however, these functions are yet to be determined. The latter possibility seems plausible in view of a recent publication that implicates miR-146a in the suppression of NO production in macrophages (Li et al., 2016). Initially, BCG mediated upregulation of miR-146a was attributed only to the alteration of cytokines like TNF-α (Li et al., 2013), but it's role in the suppression of NO has only recently been established (Li et al., 2016). Lastly, considering the fact that the miRNA field is still evolving and that miRNAs have multiple mRNA targets, assessing the potential interactions between altered miRNAs with the mRNAs of proteins associated with specific bactericidal pathways may help shed light on this issue.

It is very encouraging to note that studies aimed at understanding the roles of miRNAs in bacterial infections have been gaining momentum in recent years. Invariably, in many of these studies, selected cell types were used for miRNA profiling, even if the cells were derived from infected animals or humans. Since miRNAs are expressed in a tissue and cell specific manner, identifying miRNAs in a selected cell type limits our ability to obtain a comprehensive picture of miRNAs altered by a specific pathogen inside the host. Further, there have been recent reports that gut commensal bacteria can influence the miRNA profiles to a specific pathogen (Archambaud et al., 2013). This is a concern as it complicates the interpretation of previous findings. Despite these issues, studies on miRNAs associated with intracellular bacteria and other pathogens are likely to contribute to the development of therapeutics and biomarkers, especially for those that cause chronic and deadly diseases like *M. tuberculosis*.

## Author contributions

KD: Collected data, created the tables. OG: Assisted in writing the manuscript provided critical comments. SD: Conceived and designed the study, wrote the manuscript.

## Funding

This study was supported by grants from NIH R21AI097913, Robert J. Kleberg, Jr. and Helen C. Kleberg Foundation and TTUHSCEP seed grant program.

### Conflict of interest statement

The authors declare that the research was conducted in the absence of any commercial or financial relationships that could be construed as a potential conflict of interest.
